# Supplementary material for: “Targeting NF-κB epigenetic activation and DNA repair deficiency in G34-mutant pediatric diffuse hemispheric glioma with nanoparticles combining PARP inhibition and immune stimulation mediated by CpG dinucleotides”
Source: bioRxiv. 2026 Mar 11:2026.03.09.710503. Preprint. [Version 1] doi: 10.64898/2026.03.09.710503 (PMC13060902; doi:10.64898/2026.03.09.710503)
Supplement: Supplement 4 [file NIHPP2026.03.09.710503v1-supplement-4.pdf]

bioRxiv preprint doi: https://doi.org/10.64898/2026.03.09.710503; this version posted March 11, 2026. The copyright holder for this preprint (which was not certified by peer review) is the author/funder, who has granted bioRxiv a license to display the preprint in perpetuity. It is made available under aCC-BY 4.0 International license.

Supplementary Figures

bioRxiv preprint doi: https://doi.org/10.64898/2026.03.09.710503; this version posted March 11, 2026. The copyright holder for this preprint (which was not certified by peer review) is the author/funder, who has granted bioRxiv a license to display the preprint in perpetuity. It is made available under aCC-BY 4.0 International license.

Figure S1 - NF-κB gene expression network in DHG cells

Mouse DHG cells (*in vivo*) G34R-mutant vs histone Wt

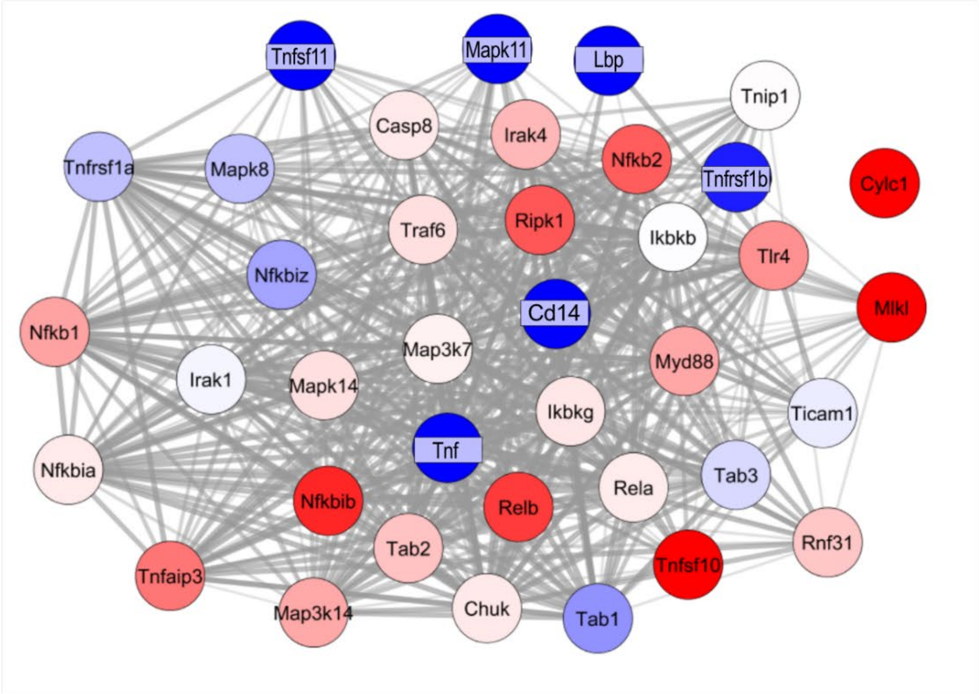

Human DHG cells (*in vitro*) G34R-mutant vs histone Wt

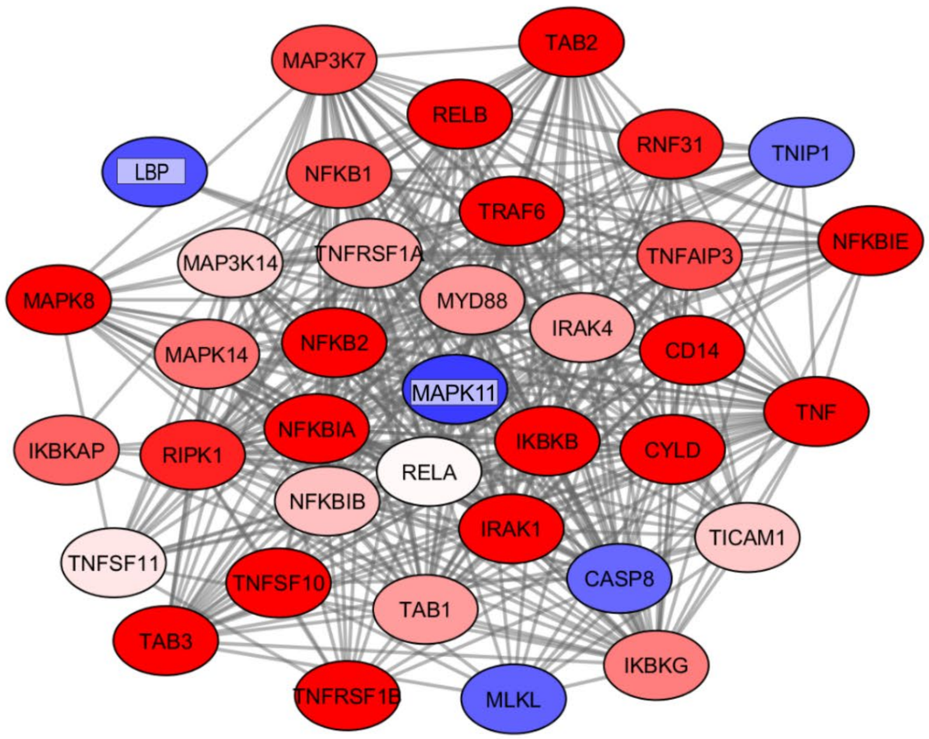

bioRxiv preprint doi: https://doi.org/10.64898/2026.03.09.710503; this version posted March 11, 2026. The copyright holder for this preprint (which was not certified by peer review) is the author/funder, who has granted bioRxiv a license to display the preprint in perpetuity. It is made available under aCC-BY 4.0 International license.

Supplementary Figure 1. Transcriptional expression of NF-κB-related genes in G34R-mutant versus histone Wt mouse and human DHG cells.

bioRxiv preprint doi: https://doi.org/10.64898/2026.03.09.710503; this version posted March 11, 2026. The copyright holder for this preprint (which was not certified by peer review) is the author/funder, who has granted bioRxiv a license to display the preprint in perpetuity. It is made available under aCC-BY 4.0 International license.

Supplementary Figure 2: DNA repair and cell cycle postraslational activation in G34R mouse DHG cells after treatment with olaparib-sHDL-CpG nanoparticles

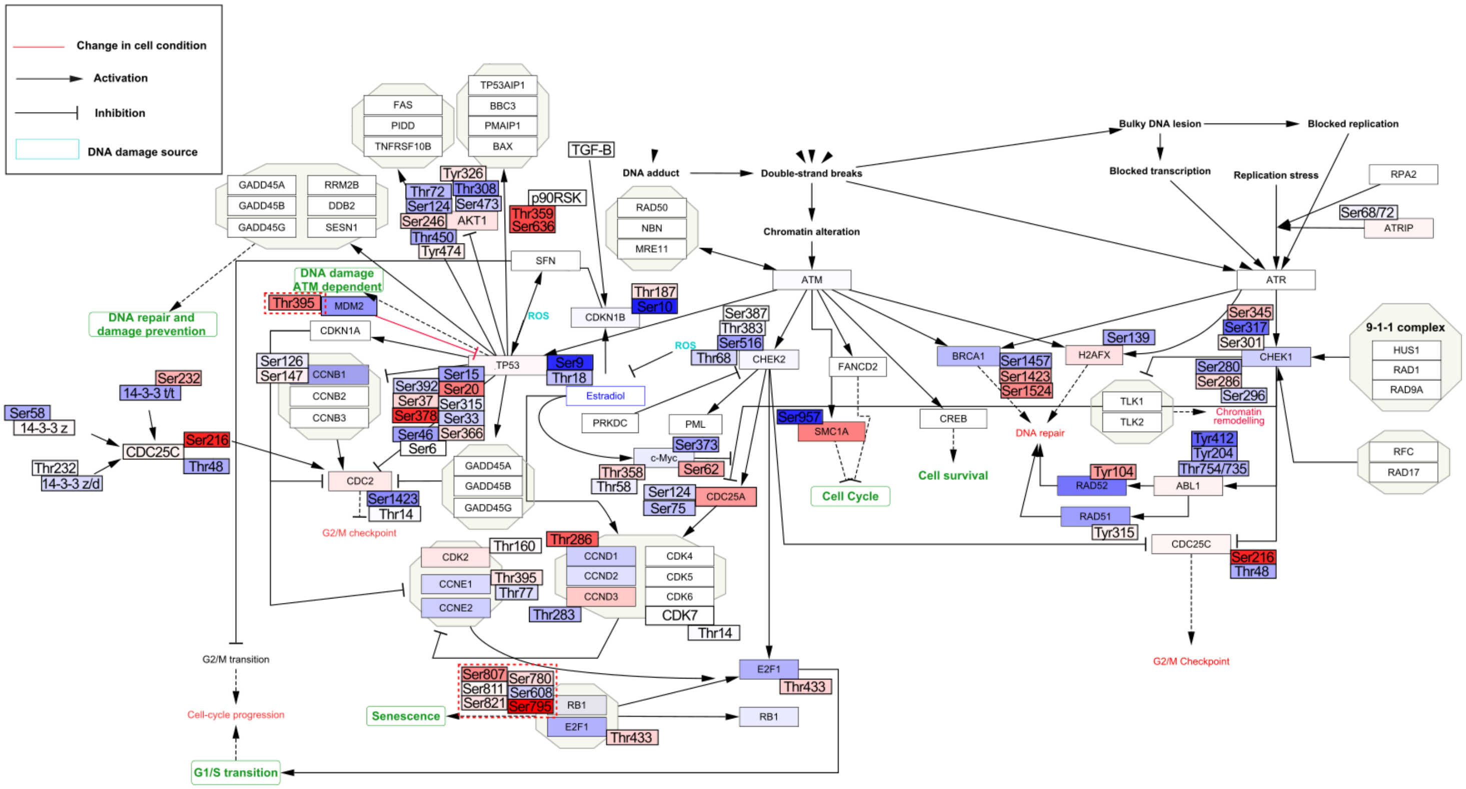

bioRxiv preprint doi: https://doi.org/10.64898/2026.03.09.710503; this version posted March 11, 2026. The copyright holder for this preprint (which was not certified by peer review) is the author/funder, who has granted bioRxiv a license to display the preprint in perpetuity. It is made available under aCC-BY 4.0 International license.

Supplementary figure 2. Postraslational activation of DNA repair and cell cycle genes in G34R mouse pHGG cells after treatment with olaparib-sHDL-CpG nanoparticles

bioRxiv preprint doi: https://doi.org/10.64898/2026.03.09.710503; this version posted March 11, 2026. The copyright holder for this preprint (which was not certified by peer review) is the author/funder, who has granted bioRxiv a license to display the preprint in perpetuity. It is made available under aCC-BY 4.0 International license.

NF-κB activation in G34R-mutant DHG cells upon stimulation with sHDL-CpG

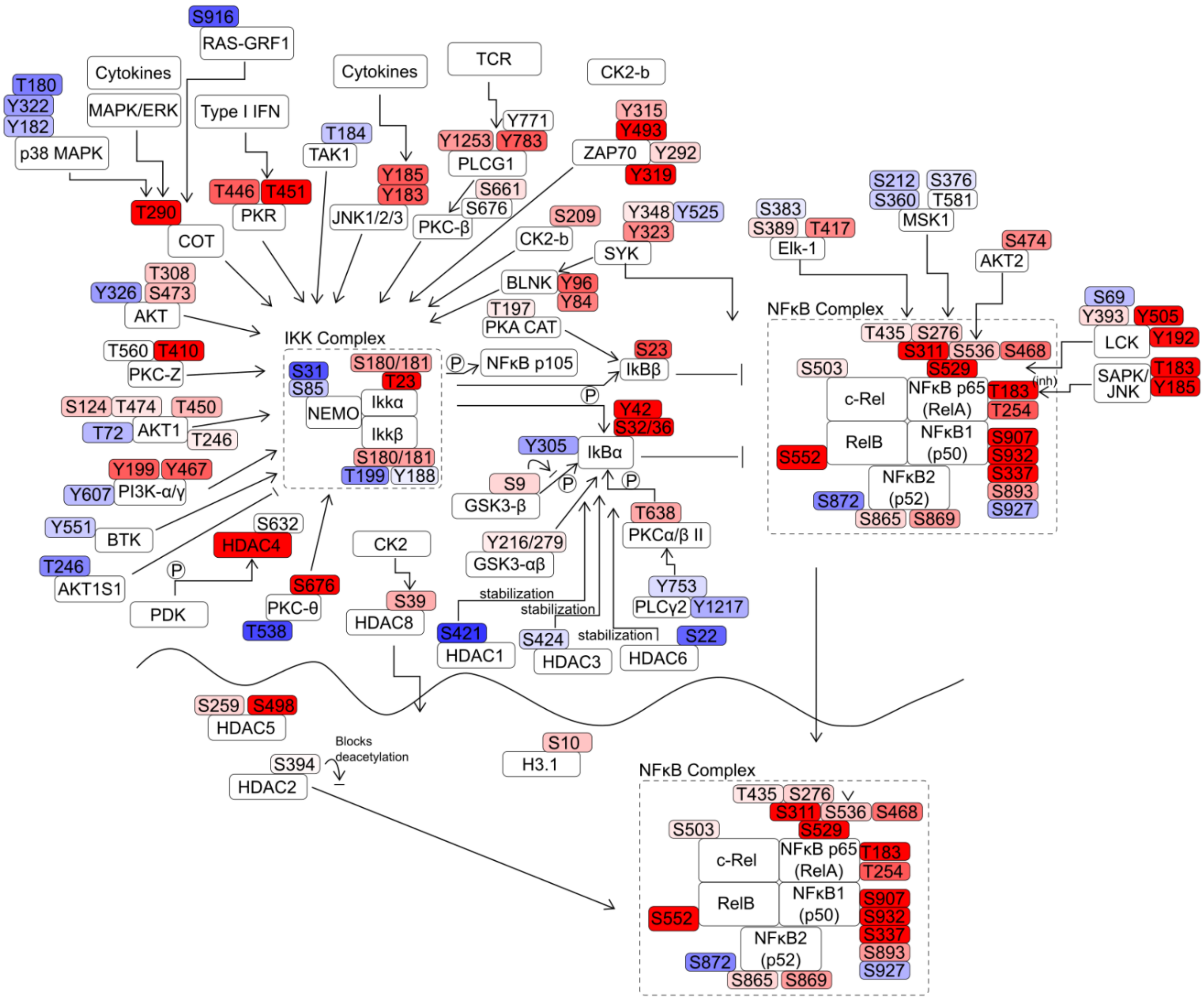

bioRxiv preprint doi: https://doi.org/10.64898/2026.03.09.710503; this version posted March 11, 2026. The copyright holder for this preprint (which was not certified by peer review) is the author/funder, who has granted bioRxiv a license to display the preprint in perpetuity. It is made available under aCC-BY 4.0 International license.

Supplementary Figure 3. Postraslational activation of NF-kB genes in G34R-mutant DHG cells upon stimulation with sHDL-CpG nanoparticles

bioRxiv preprint doi: https://doi.org/10.64898/2026.03.09.710503; this version posted March 11, 2026. The copyright holder for this preprint (which was not certified by peer review) is the author/funder, who has granted bioRxiv a license to display the preprint in perpetuity. It is made available under aCC-BY 4.0 International license.

Cytokine CCL5 and CXCL10 levels in response to Olaparib-sHDL-CpG nanoparticles and NF-κB inhibitors in H3.3-Wt DHG cells

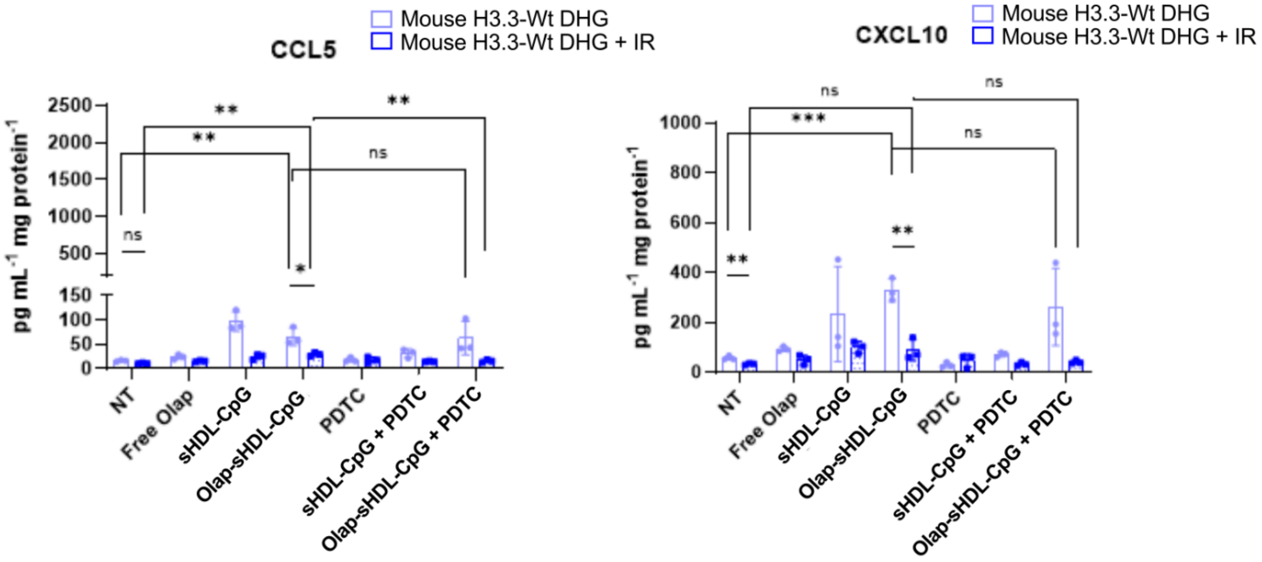

bioRxiv preprint doi: https://doi.org/10.64898/2026.03.09.710503; this version posted March 11, 2026. The copyright holder for this preprint (which was not certified by peer review) is the author/funder, who has granted bioRxiv a license to display the preprint in perpetuity. It is made available under aCC-BY 4.0 International license.

Supplementary Figure 4. CCL5 and CXCL10 cytokine levels on H3.3-Wt mouse DHG cells upon stimulation with olaparib-sHDL-CpG nanoparticles in presence or absence of NF-κB inhibitors.

bioRxiv preprint doi: https://doi.org/10.64898/2026.03.09.710503; this version posted March 11, 2026. The copyright holder for this preprint (which was not certified by peer review) is the author/funder, who has granted bioRxiv a license to display the preprint in perpetuity. It is made available under aCC-BY 4.0 International license.

Cytokine levels in response to olaparib-sHDL-CpG nanoparticles and NF-κB inhibitors

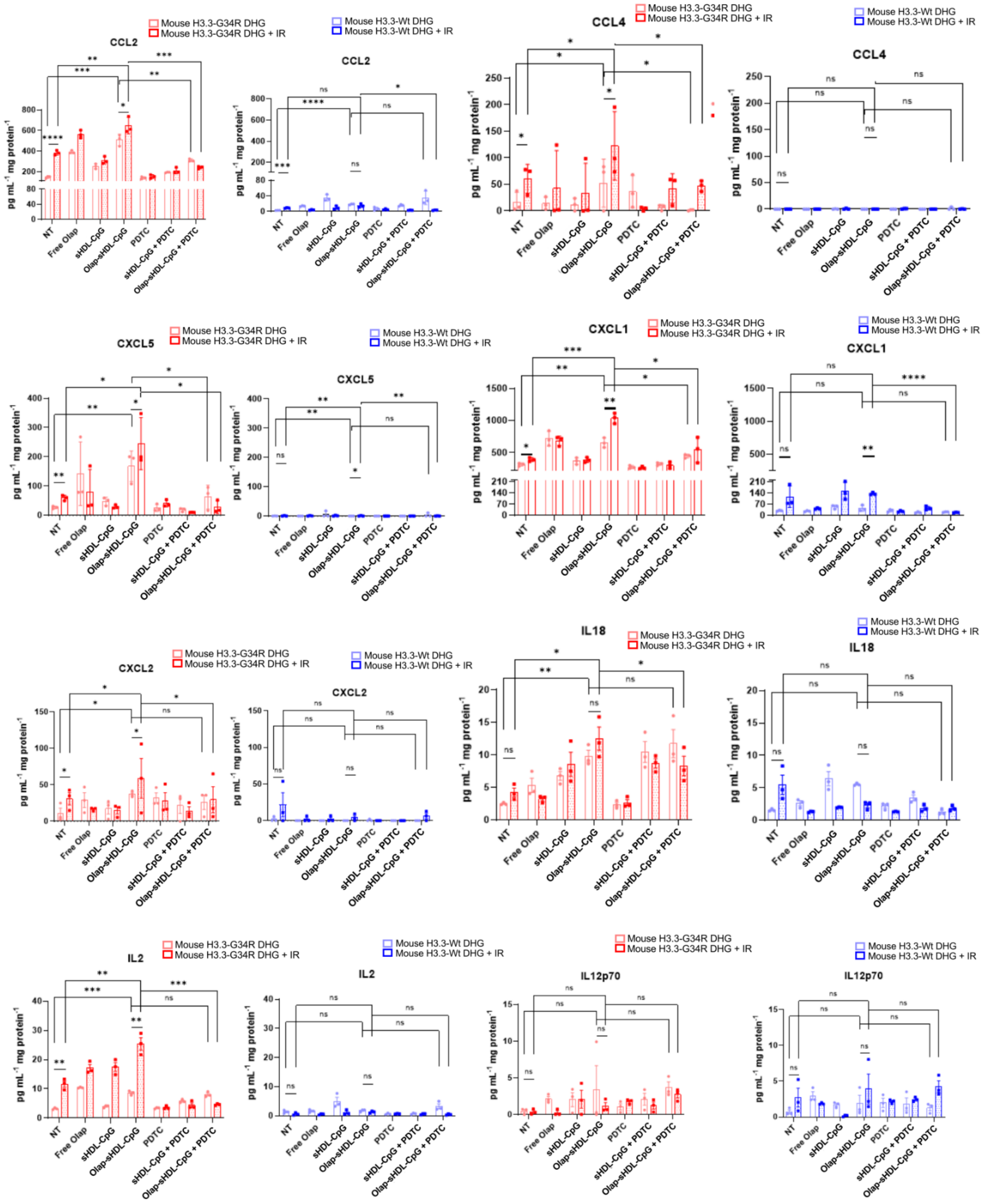

bioRxiv preprint doi: https://doi.org/10.64898/2026.03.09.710503; this version posted March 11, 2026. The copyright holder for this preprint (which was not certified by peer review) is the author/funder, who has granted bioRxiv a license to display the preprint in perpetuity. It is made available under aCC-BY 4.0 International license.

Supplementary Figure 5. Cytokine levels on G34R-mutant and H3.3-Wt mouse DHG cells upon stimulation with olaparib-sHDL-CpG nanoparticles in presence or absence of NF-κB inhibitors.

bioRxiv preprint doi: https://doi.org/10.64898/2026.03.09.710503; this version posted March 11, 2026. The copyright holder for this preprint (which was not certified by peer review) is the author/funder, who has granted bioRxiv a license to display the preprint in perpetuity. It is made available under aCC-BY 4.0 International license.

DAMPs HMGB1 and TNF-α levels in response to Olaparib-sHDL-CpG nanoparticles and NF-κB inhibitors in H3.3-Wt DHG cells

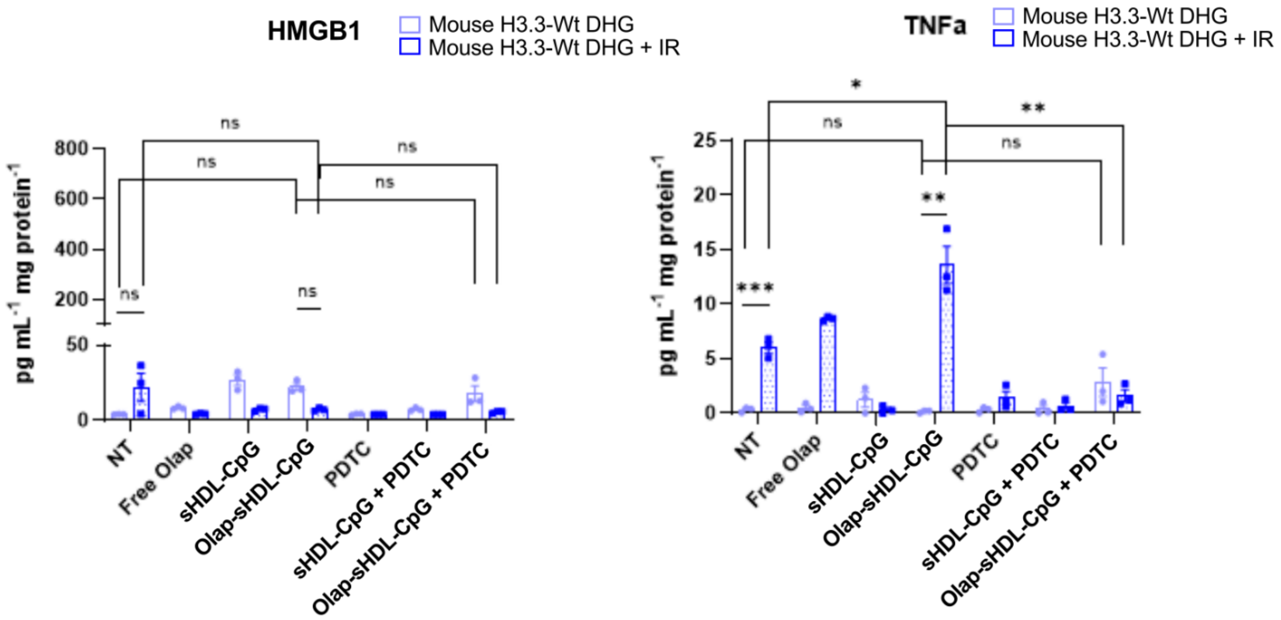

bioRxiv preprint doi: https://doi.org/10.64898/2026.03.09.710503; this version posted March 11, 2026. The copyright holder for this preprint (which was not certified by peer review) is the author/funder, who has granted bioRxiv a license to display the preprint in perpetuity. It is made available under aCC-BY 4.0 International license.

Supplementary Figure 6. HMGB1 and TNFα DAMPs levels on H3.3-Wt mouse DHG cells upon stimulation with olaparib-sHDL-CpG nanoparticles in presence or absence of NF-κB inhibitors.

bioRxiv preprint doi: https://doi.org/10.64898/2026.03.09.710503; this version posted March 11, 2026. The copyright holder for this preprint (which was not certified by peer review) is the author/funder, who has granted bioRxiv a license to display the preprint in perpetuity. It is made available under aCC-BY 4.0 International license.

DAMPs levels in response to olaparib-sHDL-CpG nanoparticles and NF-κB inhibitors

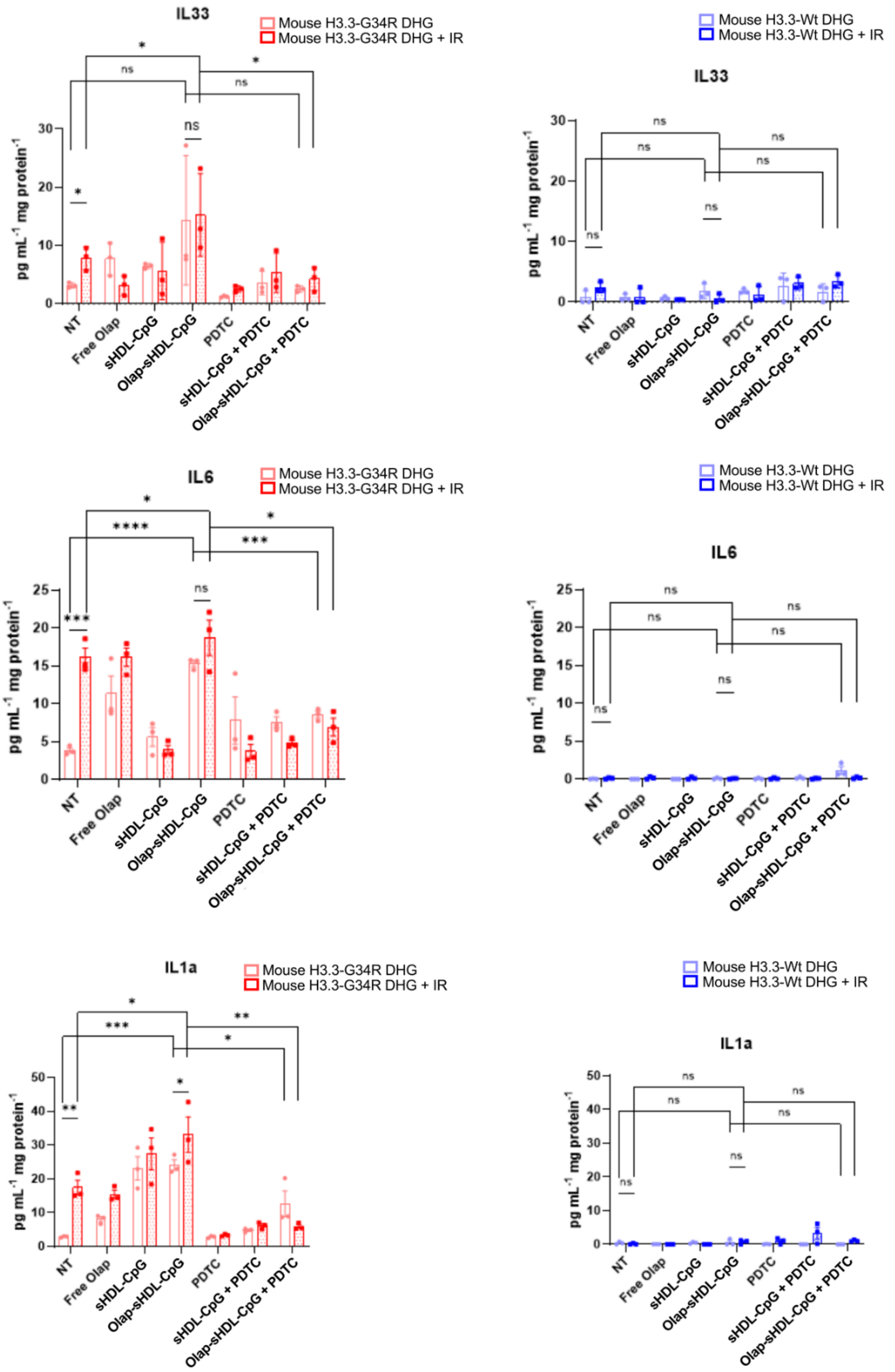

bioRxiv preprint doi: https://doi.org/10.64898/2026.03.09.710503; this version posted March 11, 2026. The copyright holder for this preprint (which was not certified by peer review) is the author/funder, who has granted bioRxiv a license to display the preprint in perpetuity. It is made available under aCC-BY 4.0 International license.

Supplementary Figure 7. HMGB1 and TNFα DAMPs levels on G34R-mutant and H3.3-Wt mouse DHG cells upon stimulation with olaparib-sHDL-CpG nanoparticles in presence or absence of NF-κB inhibitors.

bioRxiv preprint doi: https://doi.org/10.64898/2026.03.09.710503; this version posted March 11, 2026. The copyright holder for this preprint (which was not certified by peer review) is the author/funder, who has granted bioRxiv a license to display the preprint in perpetuity. It is made available under aCC-BY 4.0 International license.

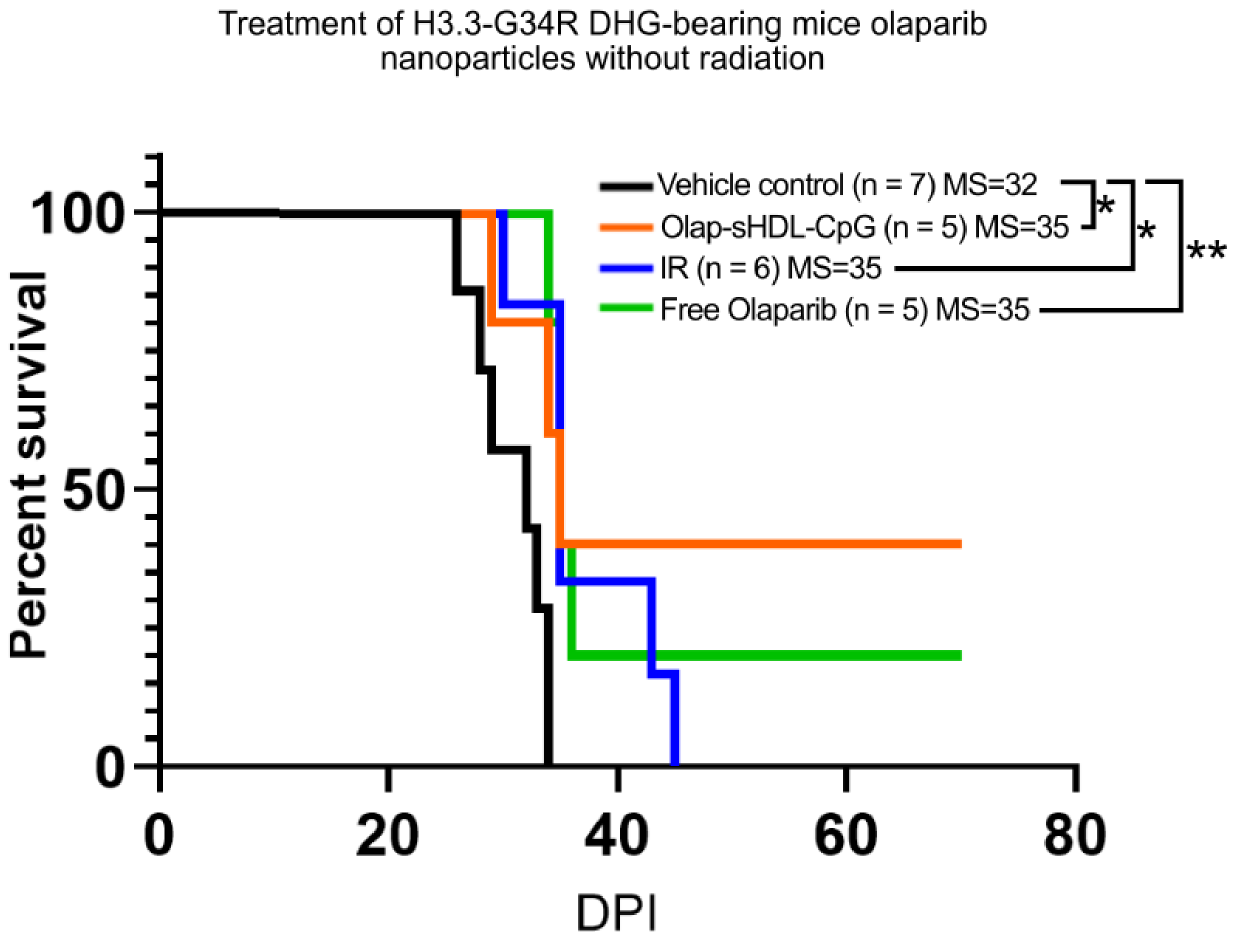

bioRxiv preprint doi: https://doi.org/10.64898/2026.03.09.710503; this version posted March 11, 2026. The copyright holder for this preprint (which was not certified by peer review) is the author/funder, who has granted bioRxiv a license to display the preprint in perpetuity. It is made available under aCC-BY 4.0 International license.

Supplementary Figure 8. Survival curves H3.3-G34R DHG-bearing mice treated with olaparib-sHDL-CpG nanoparticles or free olaparib without radiation.

bioRxiv preprint doi: https://doi.org/10.64898/2026.03.09.710503; this version posted March 11, 2026. The copyright holder for this preprint (which was not certified by peer review) is the author/funder, who has granted bioRxiv a license to display the preprint in perpetuity. It is made available under aCC-BY 4.0 International license.

Complete serum chemestry of mice bearing G34-mutant DHG tumors upon Olaparib-sHDL-CpG treatment

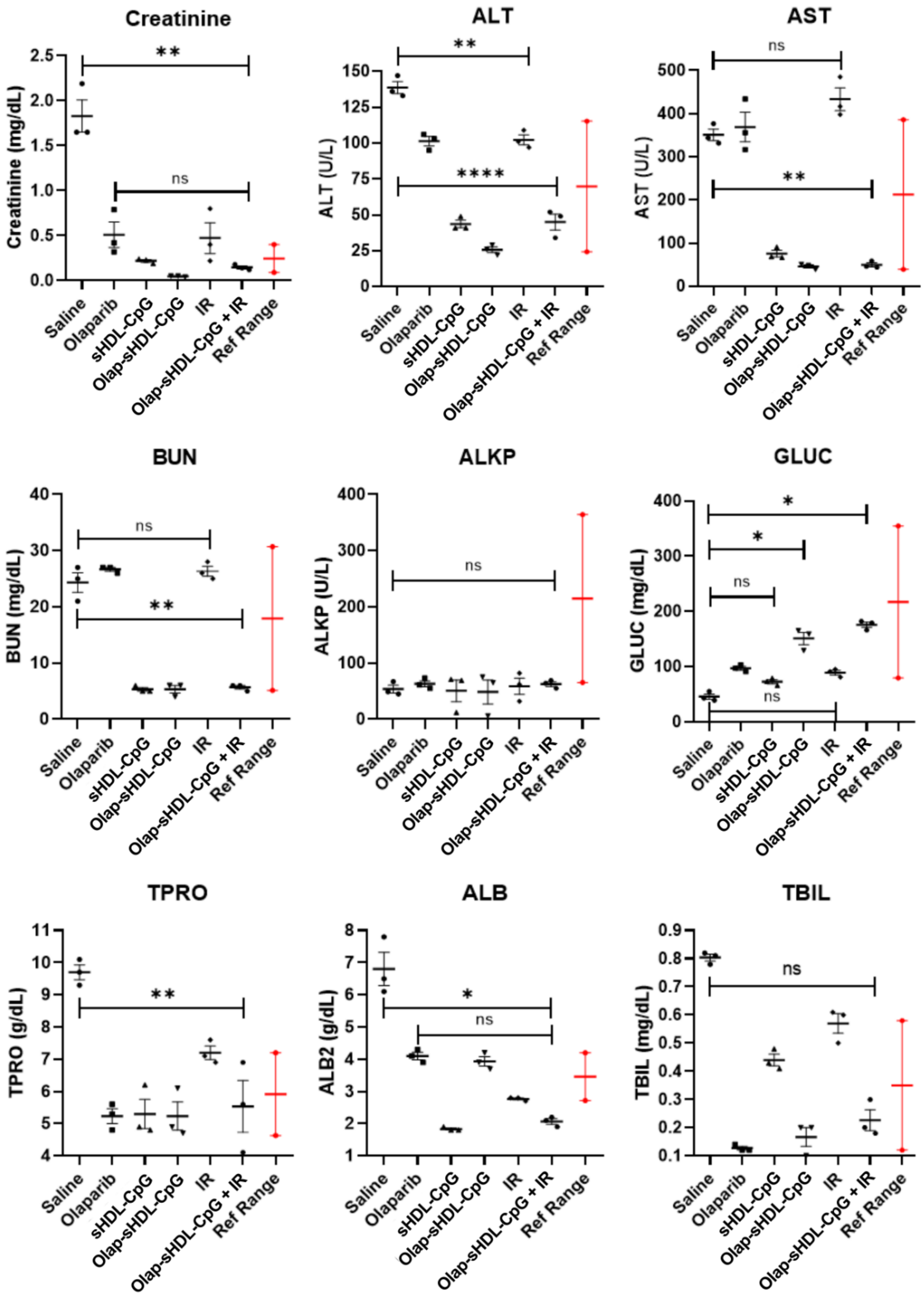

bioRxiv preprint doi: https://doi.org/10.64898/2026.03.09.710503; this version posted March 11, 2026. The copyright holder for this preprint (which was not certified by peer review) is the author/funder, who has granted bioRxiv a license to display the preprint in perpetuity. It is made available under aCC-BY 4.0 International license.

Supplementary Figure 9. Assessment of liver function via evaluation of serum chemistry markers on H3.3-G34R DHG-bearing mice treated with olaparib-sHDL-CpG nanoparticles.

bioRxiv preprint doi: https://doi.org/10.64898/2026.03.09.710503; this version posted March 11, 2026. The copyright holder for this preprint (which was not certified by peer review) is the author/funder, who has granted bioRxiv a license to display the preprint in perpetuity. It is made available under aCC-BY 4.0 International license.

Histological and immunohistochemical analysis of mouse brain after saline, irradiation, free olaparib, and Olaparib-sHDL-CpG combination treatments

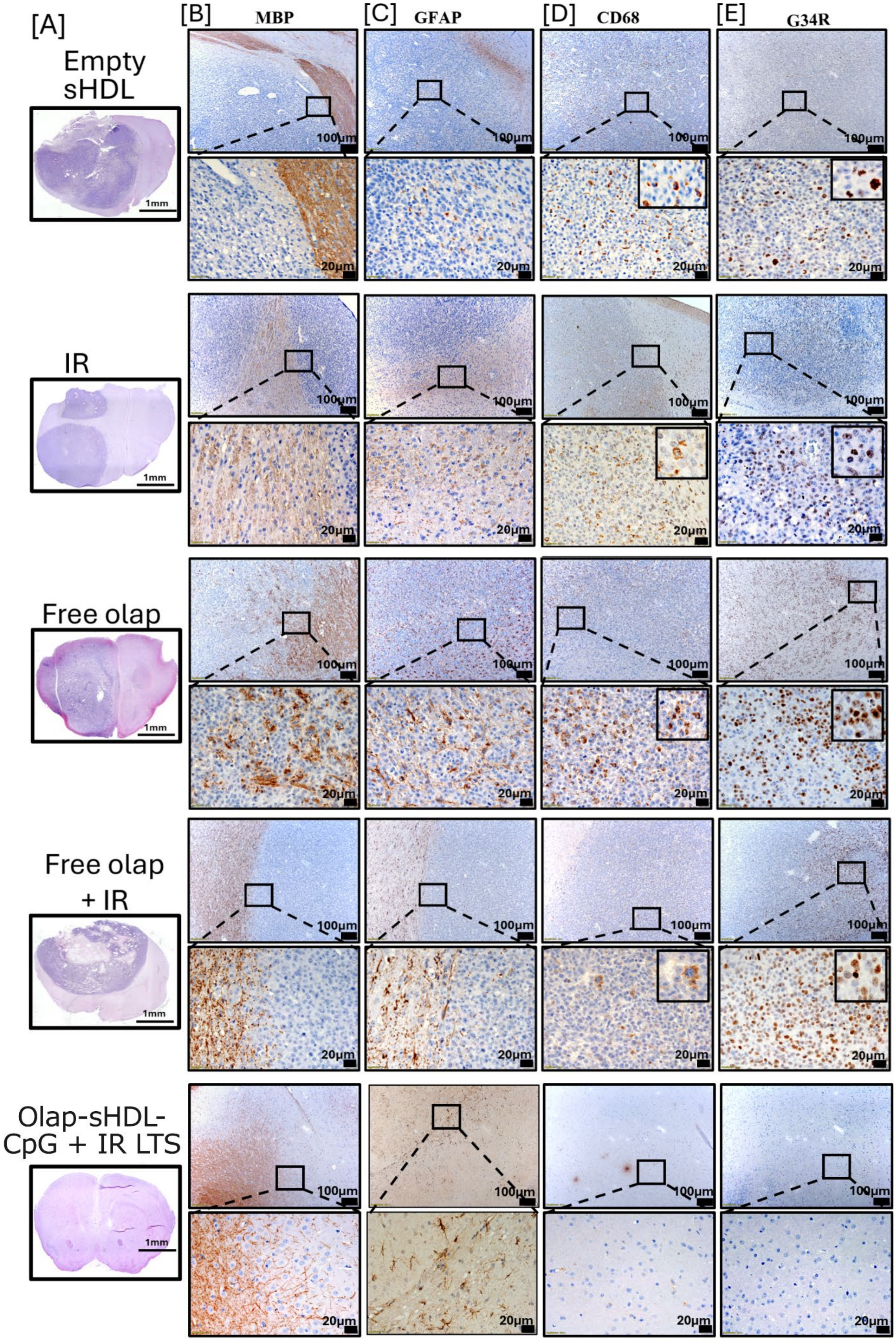

bioRxiv preprint doi: https://doi.org/10.64898/2026.03.09.710503; this version posted March 11, 2026. The copyright holder for this preprint (which was not certified by peer review) is the author/funder, who has granted bioRxiv a license to display the preprint in perpetuity. It is made available under aCC-BY 4.0 International license.

Supplementary Figure 10. IHC tumor microenvironment and brain inflammation analysis on brains of H3.3-G34R DHG-bearing mice treated with olaparib-sHDL-CpG nanoparticles. Treatment group is indicated in (A) and antibody stains are indicated in (B) (MBP = Myelin basic protein), (C) (GFAP = glial fibrillary acidic protein), (D) (CD68 = CD68 glycoprotein, primarily expressed on macrophages and monocytes), and (E) (G34R= Anti H3.3 histone G34R-mutant)

bioRxiv preprint doi: https://doi.org/10.64898/2026.03.09.710503; this version posted March 11, 2026. The copyright holder for this preprint (which was not certified by peer review) is the author/funder, who has granted bioRxiv a license to display the preprint in perpetuity. It is made available under aCC-BY 4.0 International license.

Histological and immunohistochemical analysis of mouse brain after saline, irradiation, free olaparib, and Olaparib-sHDL-CpG combination treatments - immune markers

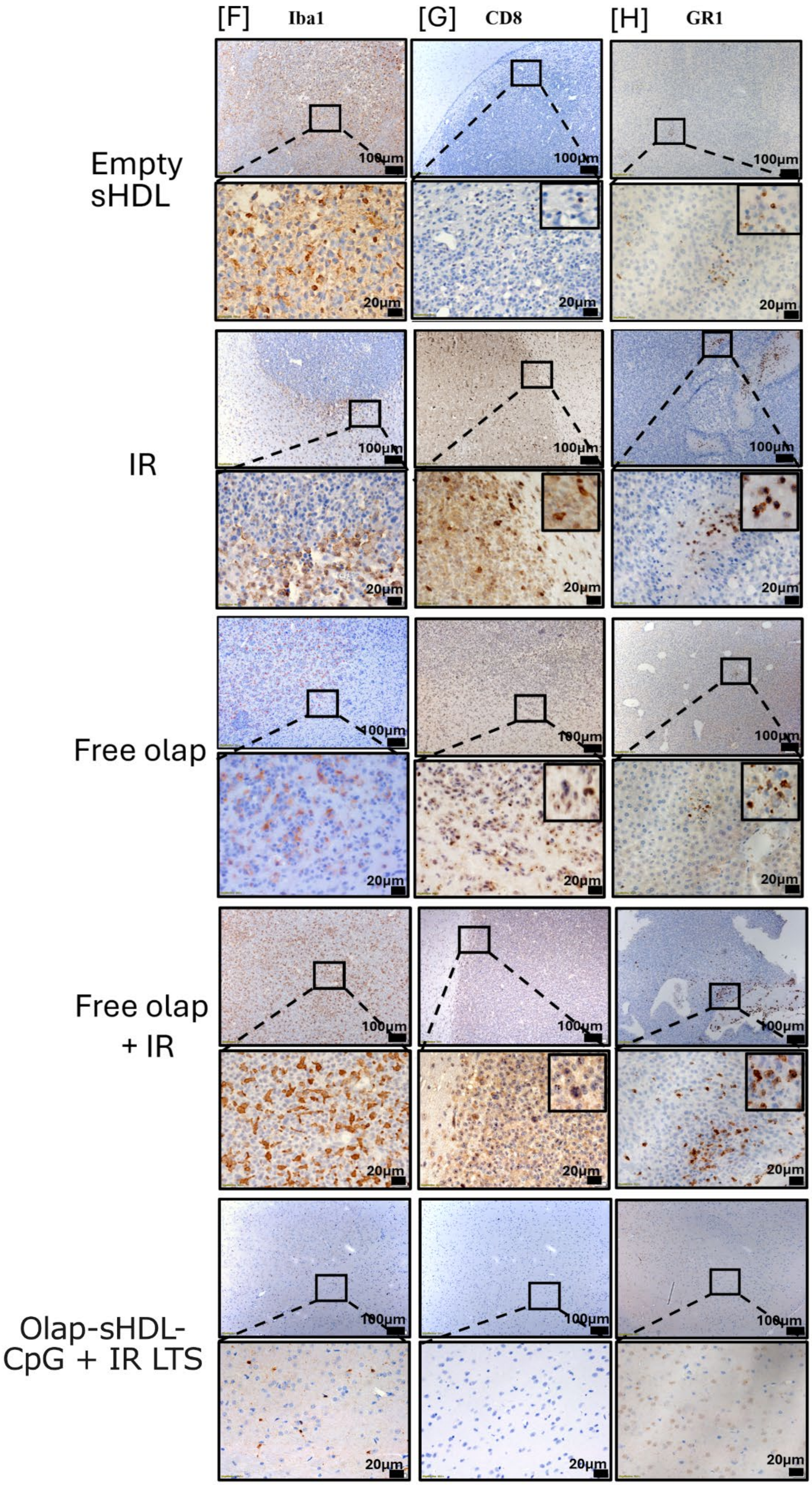

bioRxiv preprint doi: https://doi.org/10.64898/2026.03.09.710503; this version posted March 11, 2026. The copyright holder for this preprint (which was not certified by peer review) is the author/funder, who has granted bioRxiv a license to display the preprint in perpetuity. It is made available under aCC-BY 4.0 International license.

Supplementary Figure 11. IHC tumor microenvironment and brain inflammation analysis on brains of H3.3-G34R DHG-bearing mice treated with olaparib-sHDL-CpG nanoparticles. Antibody stains are indicated in (A) (IBA1 = ionized calcium-binding adapter molecule 1, marker for microglia and macrophages), (B) , (CD8 = CD8 antigen, a cell-surface glycoprotein primarily found on cytotoxic T-lymphocytes (CTLs)), and (C) (Gr1 = targets the Ly-6G antigen, a myeloid differentiation marker primarily found on neutrophils, monocytes, and granulocytes).
